# Improved therapeutic effects on diabetic foot by human mesenchymal stem cells expressing MALAT1 as a sponge for microRNA-205-5p

**DOI:** 10.18632/aging.102562

**Published:** 2019-12-21

**Authors:** Lingyan Zhu, Qiaoqing Zhong, Tianlun Yang, Xiangwei Xiao

**Affiliations:** 1 Department of Endocrinology, The First Affiliated Hospital of Nanchang University, Nanchang 330006, China; 2 Department of Cardiology, Xiangya Hospital, Central South University, Changsha 410078, China; 3 National Clinical Research Center for Geriatric Disorders, Xiangya Hospital, Central South University, Changsha 410008, China; 4 Division of Pediatric Surgery, Department of Surgery, Children’s Hospital of Pittsburgh, University of Pittsburgh School of Medicine, Pittsburgh, PA 15224, USA

**Keywords:** diabetic foot, MALAT1, mesenchymal stem cells, microRNA, VEGF

## Abstract

Diabetic foot (DF) is a common complication of high severity for diabetes, a prevalent metabolic disorder that affects billions of people worldwide. Mesenchymal stem cells (MSCs) have a demonstrative therapeutic effect on DF, through their generation of pro-angiogenesis factors, like vascular endothelial growth factor (VEGF). Recently, genetically modified MSCs have been used in therapy and we have shown that depletion of micoRNA-205-5p (miR-205-5p) in human MSCs promotes VEGF-mediated therapeutic effects on DF. Here, we showed that a long non-coding RNA (lncRNA), MALAT1, is a competing endogenous RNA (ceRNA) for miR-205-5p, and is low expressed in human MSCs. Ectopic expression of MALAT1 in human MSCs significantly decreased miR-205-5p levels, resulting in upregulation of VEGF production and improved in vitro endothelial cell tube formation. In a DF model in immunodeficient NOD/SCID mice, transplantation of human miR-205-5p-depleted MSCs exhibited better therapeutic effects on DF recovery than control MSCs. Moreover, MALAT1-expressing MSCs showed even better therapeutic effects on DF recovery than miR-205-5p-depleted MSCs. This difference in DF recovery was shown to be associated with the levels of on-site vascularization. Together, our data suggest that MALAT1 functions as a sponge RNA for miR-205-5p to increase therapeutic effects of MSCs on DF.

## INTRODUCTION

Mesenchymal stem cells (MSCs) have a demonstrative therapeutic effect on diabetic foot (DF), through their differentiation into endothelial cells and their generation of pro-angiogenesis factors, like vascular endothelial growth factor (VEGF), to promote vascularization in the sick foot. Recently, genetically modified MSCs have been used in therapy and we have shown that depletion of micoRNA-205-5p (miR-205-5p) in human MSCs promotes their secretion of VEGF to increase their therapeutic effects on DF, through augmentation of VEGF-mediated vascularization.

DF is a common complication of high severity for diabetes, is a prevalent metabolic disorder that affects billions of people worldwide [1]. The pathogenesis of DF stems from the alteration of angiogenesis, functionality of immune cells, homeostasis of extracellular matrix and fibrogenesis due to hyperglycemic status [2]. Previous therapeutic approaches and researches have highlighted revascularization as a key strategy for treating DF, and VEGF is the most important pro-angiogenesis factor that exerts critical effects on the recovery of DF [3]. In the family of VEGF, VEGF-A is the most potent pro-angiogenic factor, and thus is simplified as VEGF in the scope of the current study. Our previous studies have shown the importance of VEGF in the biology of pancreatic beta cells and duct cells [4, 5].

Bone marrow-derived MSCs are have multipotent differential potentials [6]. MSCs have been commonly used in treatments promoting tissue repair, including DF, due to their high accessibility, easy expansion ex vivo and multiple differentiation potentials [7]. Recent studies have shown that epigenetics of MSCs are not optimal for certain therapy and thus modification of MSCs with specific genes for specific aims are applied to further improve the therapeutic potentials of MSCs [8–10]. Alongside with these discoveries, we disclosed that depletion of miR-205-5p in human MSCs promotes their secretion of VEGF to increase their therapeutic effects on DF, through augmentation of VEGF-mediated vascularization [11]. These studies all contribute to our understanding of genetic modification on MSCs-based gene therapy.

Different from microRNAs (miRNAs), which are non-coding small RNAs of less than 25 base pairs in length, long non-coding RNAs (lncRNAs) are longer than 200 nucleotides and lack significant protein-coding functions. Typically, lncRNAs function as competing endogenous RNAs (ceRNAs), by sponging and suppressing the expression of certain miRNAs to activate the downstream targets that are inhibited by these miRNAs [12]. Among all lncRNAs, the metastasis-associated lung adenocarcinoma transcript 1 (MALAT1), is a relatively well studied one. MALAT1 is located within human chromosome 11q13.1 and the primary sequence of MALAT1 gene contains about 8000 bp and displays a high level of conservation throughout 33 mammalian species [13]. MALAT1 is a single exon gene and lacks of open reading frames of significant length, which is necessary for protein synthesis [13]. Some recent studies have highlighted MALAT1 as a sponge lncRNA for miR-205 in cells including renal carcinoma [14], osteosarcoma [15], and neuronal cells [16]. However, the relation between MALAT1 and miR-205-5p in MSCs and its related application in treating DF have not been reported.

Here, we showed that MALAT1 is a ceRNA for miR-205-5p, and is low expressed in human MSCs. Ectopic expression of MALAT1 in human MSCs significantly decreased miR-205-5p levels, resulting in upregulation of VEGF production and improved in vitro endothelial cell tube formation. In a DF model generated in immunodeficient NOD/SCID mice, transplantation of human MSCs transduced with null, or with antisense of miR-205-5p (as-miR-205-5p), or MALAT1 was compared, showing better therapeutic effects on DF recovery by MALAT1 overexpression than by miR-205-5p depletion, which seemed to be associated with improved vascularization on the disease site.

## RESULTS

### MALAT1 is a ceRNA for miR-205-5p, and is low expressed in human MSCs

**Figure 1:**
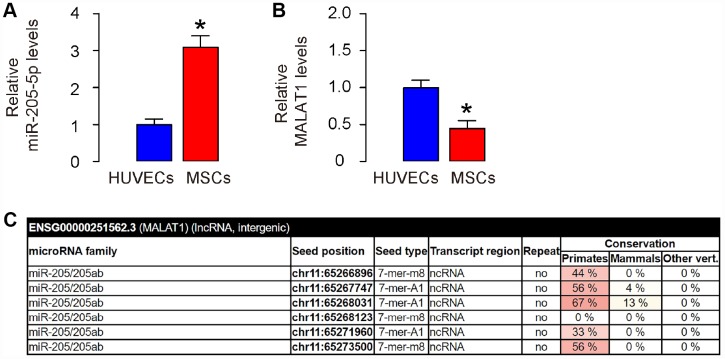
**MALAT1 is a ceRNA for miR-205-5p, and is low expressed in human MSCs.** (**A**, **B**) Levels of miR-205-5p (**A**) and MALAT1 (**B**) were determined in human MSCs and HUVECs by RT-qPCR. (**C**) Bioinformatics prediction of binding sites on MALAT1 by miR-205-5p, showing 6 predicted binding sites, using miRcode website. *p<0.05. N=5.

The previous studies showing MALAT1 as a ceRNA for miR-205 in cells including renal carcinoma [14], osteosarcoma [15], and neuronal cells [16] inspired us to examine whether it may function similar in MSCs, since we have shown that depletion of miR-205-5p in human MSCs significantly improved its therapeutic effect on DF [11]. Thus, we compared the expression of miR-205-5p and MALAT1 in human MSCs, compared to those in human endothelial cells (HUVEC). We found that human MSCs expressed higher levels of miR-205-5p (Figure 1A), but lower levels of MALAT1 (Figure 1B), compared to HUVEC. These data are consistent with the findings in other cells showing the ceRNA characteristics of MALAT1 for miR-205 [14–16], and are consistent with our previous findings on post-transcriptional control of VEGF by miR-205-5p in human MSCs [11]. Next, we used bioinformatics analysis (http://www.mircode.org/mircode) [17] and identified 6 miR-205 binding sites on the MALAT1 sequence (Figure 1C). Together, these data suggest that MALAT1 may be a ceRNA for miR-205-5p in human MSCs, and the expression level of MALAT1 in human MSCs is low.

### Ectopic expression of MALAT1 reduces miR-205-5p and improves VEGF translation in human MSCs in vitro

**Figure 2:**
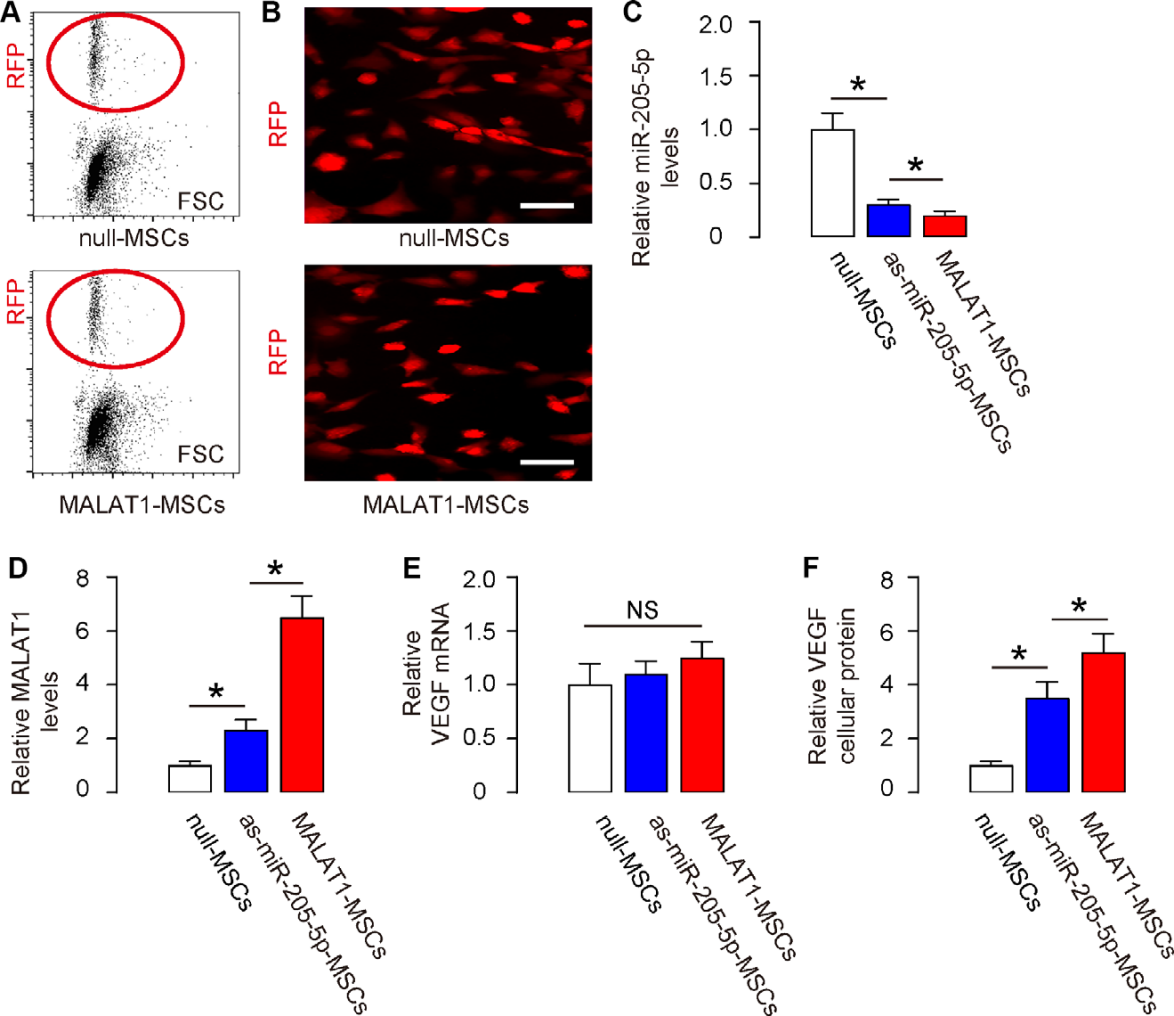
**Ectopic expression of MALAT1 reduces miR-205-5p and improves VEGF translation in human MSCs in vitro.** (**A**, **B**) Human MSCs were transduced with AAVs carrying either as-miR-205-5p, or MALAT1, or null as a control. All vectors co-expressed an RFP reporter. The transduced MSCs were purified based on RFP expression by flow cytometry (A) and exhibited red fluorescent in culture (**B**). (**C**, **E**) RT-qPCR for miR-205-5p (**C**), or MALAT1 (**D**), or VEGF (**E**) in null-, or as-miR-205-5p-, or MALAT1- transduced MSCs by RT-qPCR. (**F**) Cellular VEGF protein was determined by ELISA in null-, or as-miR-205-5p-, or MALAT1- transduced MSCs. *p<0.05. NS: non-significant. N=5. Scale bars are 20μm.

We have previously used antisense of miR-205-5p (as-miR-205-5p) to knock down miR-205-5p in human MSCs to increase VEGF production and secretion [11]. Now we sought to compare the effects of expressing MALAT1 and depleting miR-205-5p on VEGF. As previously preparing for AAV vectors carrying as-miR-205-5p [11], here we prepared MALAT1-overexpressing AAV vectors, using AAVs carrying null as controls. Both AAV vectors contained a red fluorescent protein (RFP) reporter to allow the transduced cells to be visualized under fluorescent microscopy. First, the transduced MSCs were purified based on RFP expression by flow cytometry (Figure 2A). The red fluorescence of the purified cells was readily visualized in culture (Figure 2B). The as-miR-205-5p-transduced MSCs have been described before [11]. We compared the levels of miR-205-5p, MALAT1, VEGF mRNA and cellular protein in these transduced cells with controls. We found that both miR-205-5p depletion and MALAT1 overexpression significantly reduced miR-205-5p levels in human MSCs, while the effects of MALAT1 overexpression appeared to be more pronounced (Figure 2C). On the other hand, MALAT1 overexpression dramatically and significantly increased MALAT1 levels in human MSCs, while miR-205-5p depletion also significantly increased MALAT1 levels, but to a much smaller degree (Figure 2D). Neither miR-205-5p depletion nor MALAT1 overexpression significantly altered the VEGF mRNA (Figure 2E), but both significantly increased VEGF cellular protein levels in human MSCs, while the effects of MALAT1 overexpression appeared to be more pronounced (Figure 2F). Together, these data suggest that MALAT1 appears to be a more potent miR-205-5p inhibitor and VEGF post-transcriptional activator than as-miR-205-5p in human MSCs.

### Ectopic expression of MALAT1 improves endothelial cell tube formation in vitro

**Figure 3:**
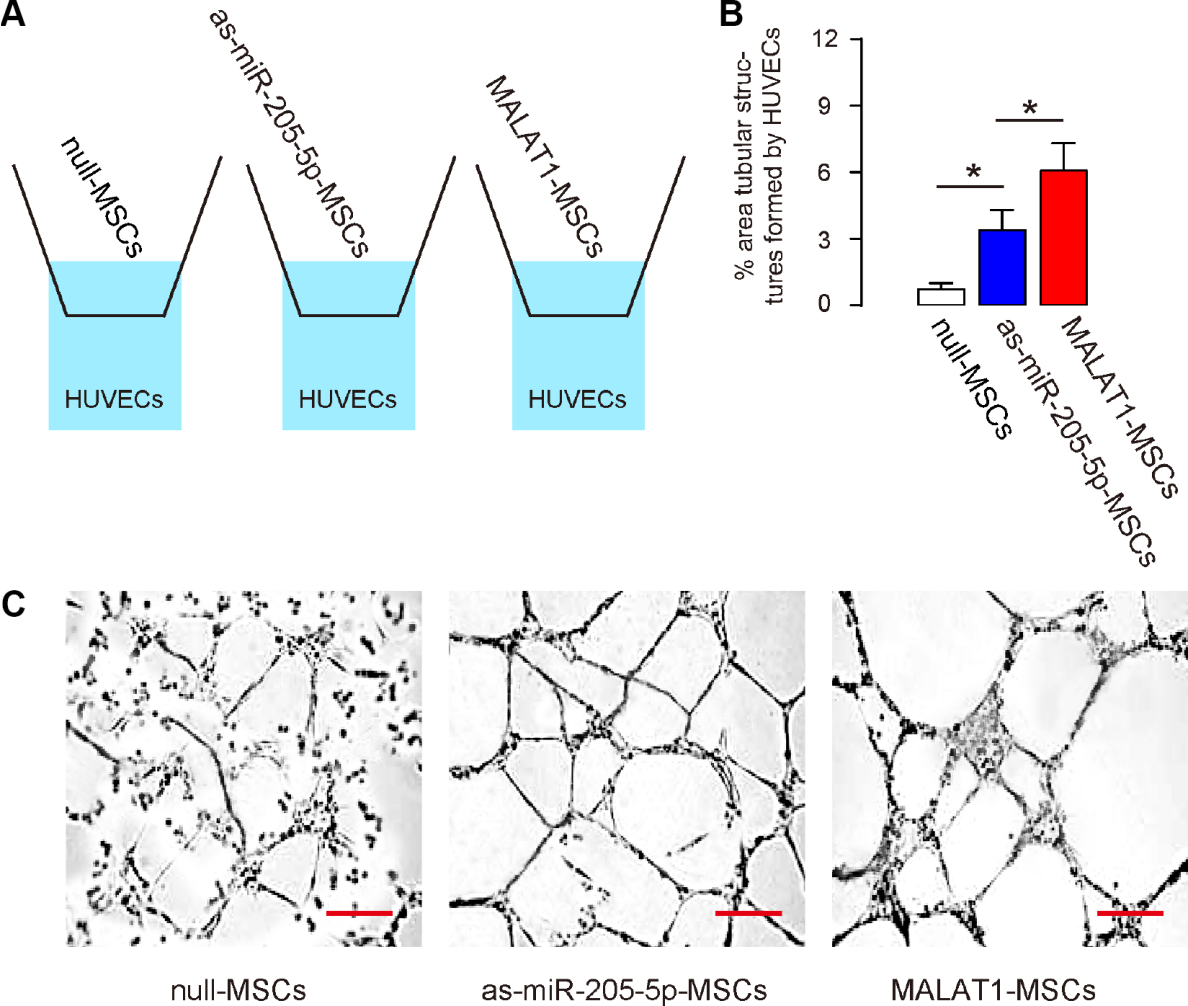
**Ectopic expression of MALAT1 improves endothelial cell tube formation in vitro.** (**A**) Schematic of a transwell co-culture system, in which HUVECs in the lower chamber were co-cultured with different MSCs (null-MSCs, as-miR-205-5p-MSCs, MALAT1-MSCs) seeded in the upper chamber. Analysis on the tube formation by HUVECs was done 3 days after co-culture. (**B**, **C**) Tube formation was assessed, shown by representative images (**B**), and by quantification (**C**). *p<0.05. N=5. Scale bars are 50μm.

In order to assess the effects of MALAT1 overexpression on MSCs-induced vascularization by endothelial cells, we used a transwell co-culture system, in which HUVECs in the lower chamber were co-cultured with different MSCs (null-MSCs, as-miR-205-5p-MSCs, MALAT1-MSCs) seeded in the upper chamber (Figure 3A). Analysis on the tube formation by HUVECs was done 3 days after co-culture, showing significant increases in tube formation of HUVECs induced by as-miR-205-5p-MSCs and MALAT1-MSCs, compared to null-MSCs, while the effects by MALAT1-MSCs were more pronounced (form more completely connected tube structures and larger area of monolayer cells, Figure 3B, 3C). Thus, ectopic expression of MALAT1 improves endothelial cell tube formation in vitro.

### Nether depletion of miR-205-5p nor MALAT1 expression in grafted MSCs reverses diabetes

**Figure 4:**
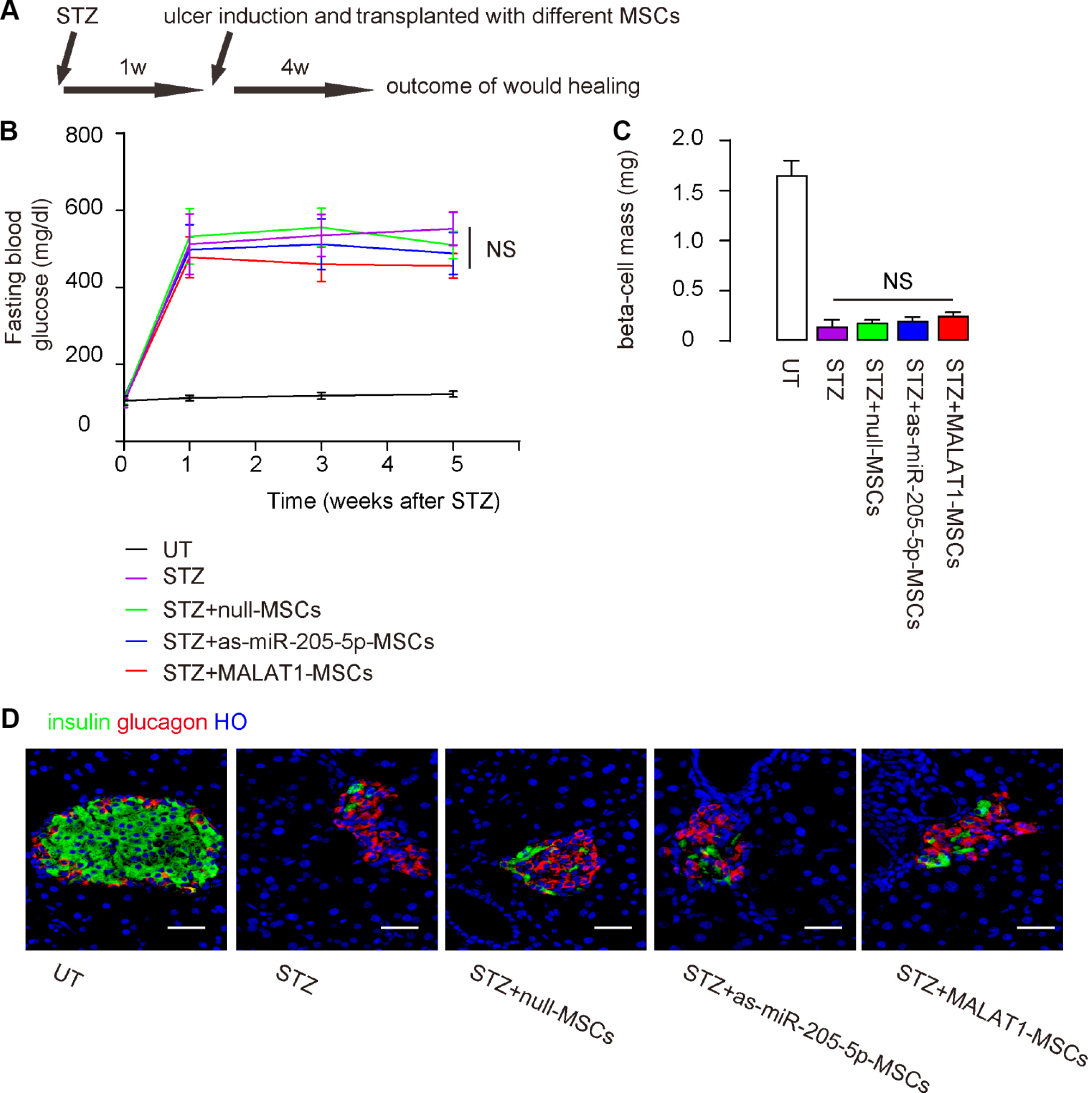
**Nether depletion of miR-205-5p nor MALAT1 overexpression in grafted MSCs reverses diabetes.** The effects of miR-205-5p-depletion or MALAT1 overexpression in MSCs on their therapeutic potential in DF were assessed. (**A**) Schematic of the model: NOD/SCID mice received STZ to develop diabetes. One week later, ulcers were generated in the right limp and the mice received on-site intradermal transplantation of either MSCs. The mice were followed up for 4 weeks before analysis. N=10 in each group. (**B**) Fasting blood glucose (**C**) Beta cell mass (**D**) Representative images for insulin immunostaining. NS: non-significant. N=10. Scale bars are 30 μm.

In order to compare the effects of miR-205-5p-depletion or MALAT1 overexpression in MSCs on their therapeutic potential in DF, the immunodeficient NOD/SCID mice received streptozotocin (STZ) to induce diabetes. One week later, ulcers were surgically made in the right limb, after which the mice received intradermal transplantation of either mull-MSCs, or as-miR-205-5p-MSCs, or MALAT1-MSCs at the ulcer site. The mice were then followed up for 4 weeks (Figure 4A). We found that STZ-treated mice developed irreversible high blood sugar in 1 week, and grafting with either type of (modified) MSCs did not correct hyperglycemia (Figure 4B), did increase beta cell mass (Figure 4C–4D). Hence, nether depletion of miR-205-5p nor MALAT1 expression in grafted MSCs reverse diabetes.

### Overexpression of MALAT1 in grafted MSCs exhibits better therapeutic potential on DF than depletion of miR-205-5p in grafted MSCs

**Figure 5:**
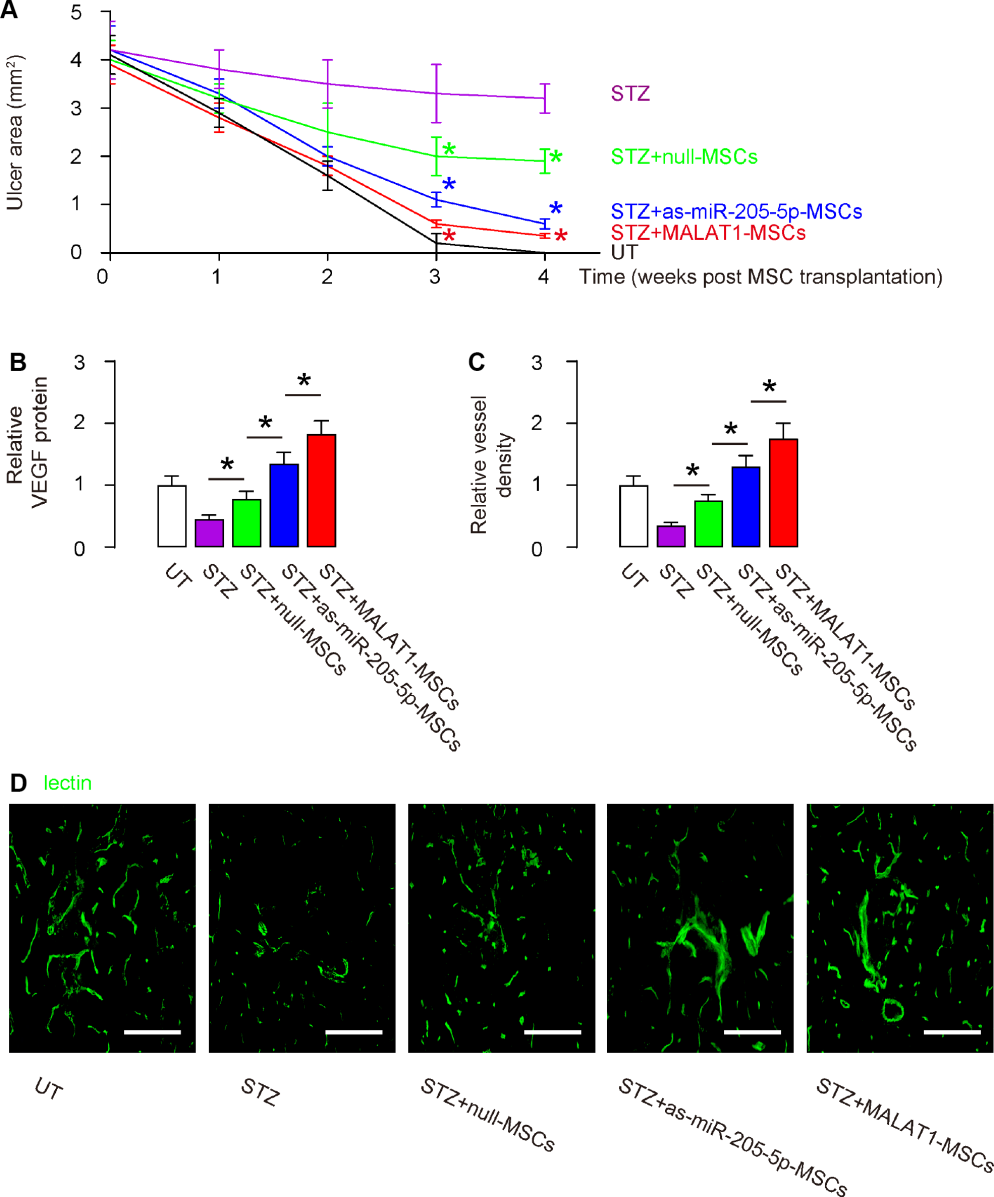
**Overexpression of MALAT1 in grafted MSCs exhibits better therapeutic potential on DF than depletion of miR-205-5p in grafted MSCs.** (**A**) Quantification of ulcer area 4 weeks after MSC transplantation. (**B**) ELISA for VEGF fold change in ulcer tissue. (**C**, **D**) Quantification of vessel density 4 weeks after MSC transplantation, shown by quantification of fold change (**C**), and by representative images (**D**). *p<0.05. N=10. Scale bars are 150 μm.

Next, we examined the effects on wound healing of DF ulcers. We found that the wound was completely cured in 4 weeks in untreated mice (UT). The ulcer did not cure at all in STZ-only treated mice. Transplantation of either (modified) MSCs improved wound healing, and the potential of therapeutic effects was the best by MALAT1-MSCs, then as-miR-205-MSCs and then null-MSCs (Figure 5A). The significant differences were detected at 3 weeks and 4 weeks after MSC transplantation (Figure 5A). VEGF protein was examined in the ulcer tissue 4 weeks after MSC transplantation and shown that more VEGF in MALAT1-MSCs-transplanted tissue, than in as-miR-205-MSCs-transplanted tissue, than in null-transplanted tissue, than STZ without transplantation (Figure 5B). Vessel density was also measured in ulcer tissue 4 weeks after MSC transplantation. We found that the vessel density was significantly reduced by STZ treatment in the ulcer tissue. Transplantation of null-MSCs significantly increased vessel density, while the increases in vessel density by transplantation of as-miR-205-5p-MSCs appeared to be greater than transplantation of null-MSCs. However, the highest increases in vessel density were detected in tissue transplanted with MALAT1-MSCs (Figure 5C–5D). Thus, overexpression of MALAT1 in grafted MSCs exhibits better therapeutic potential on DF than depletion of miR-205-5p in grafted MSCs, likely through more pronounced effects on angiogenesis.

## DISCUSSION

After discovery of MALAT1, its overexpression in many cancers has been a study of focus for many years. Different functionality and targeting genes or miRNAs by MALAT1 have been shown. Specifically, the interaction between MALAT1 and miR-205 has been demonstrated in different cell types, including renal carcinoma [14], osteosarcoma [15], and neuronal cells [16]. However, in these studies, the final effector genes by MALAT1-modifed miR-205 were not VEGF. Indeed, only we and one other group have independently shown that miR-205 is a direct regulator for VEGF protein translation, while we showed that depletion of miR-205 increased VEGF protein in MSCs to facilitate DF treatment, and the other group showed VEGF is a target of miR-205-5p in glioma [18]. In the current study, we found that overexpression of MALAT1 exhibited stronger effects on miR-205-5p and VEGF than depletion of miR-205-5p by an antisense, probably due to multiple binding sites for miR-205 on MALAT1 and the possible higher stability of MALAT1 than the antisense. Nevertheless, the better therapeutic potential on DF by MALAT1-MSCs than as-miR-205-5p appeared to present a more promising strategy to manipulate MSCs, given that MALAT1 has been extensively studied and the promoter of it has been characterized with many important regulators, including E2F4, c-Jun, c-Myc, STAT1, STAT2 and SMAD2/3 [14].

Among all VEGF family members, VEGF-A has been shown to be most potent in pro-angiogenesis and among all VEGF-A isoforms, 166 has been shown to be most effective.

It seemed that transplantation of MALAT1-MSCs has some effects on beta-cells in STZ-treated mice, although the effects may be delicate and did not reach significance in the measurement of beta-cell mass. Interestingly, it has been reported that MALAT1 is highly expressed in the pancreas [19]. Thus, MALAT1 may play critical roles in beta-cell homeostasis and function. Thus, transplanted MSCs with overexpression of MALAT1 could have some beneficial effects on the beta-cell survival or recovery from STZ treatment of on the residual beta-cell proliferation. This question may be addressed in future studies.

Although numerous studies have shown the relationship between aberrantly high expression of MALAT1 and carcinogenesis, none of these studies have reported that angiogenesis associated with VEGF transcriptional control could be an etiology. Indeed, since MALAT1 is a sponge RNA for miR-205-5p, upregulated MALAT1 is thus associated with decrease in miR-205-5p and then a subsequent increase in VEGF protein translation. High levels of VEGF may increase tumor-associated angiogenesis to promote cancer initiation and progression. Our study should provide strong evidence for related investigations on cancer.

We feel that alteration of VEGF levels in MSCs by changing its post-transcriptional regulation has several advantages than simply overexpressing VEGF in MSCs. The physiological dose of VEGF in bodies are tightly regulated in a small range, and moreover, VEGF functions very dosage-dependently [20–23]. Directly expressing VEGF may bypass many secondary regulatory pathways, which could induce robust changes in VEGF levels free of positive and negative feedback mechanisms, leading to hemorrhage or other severe problems. On the other hand, the strategies that we proposed to alter VEGF by lncRNA or miRNA are more modest, better controlled and clinically safer. Second, direct expressing VEGF in human MSCs may produce multiple excessive proteins involving in the regulatory signaling pathways, and thus lead to occurrence of ER stress. Finally, previous studies have shown adverse outcome by direct altering VEGF in patients. Together, our novel way to increase angiogenetic potential of human MSCs could be a more clinically translatable strategy.

## MATERIALS AND METHODS

### Protocol approval

Experimental protocols and methods in the current study have been approved by the research committee at the First Affiliated Hospital of Nanchang University, while animal surgery and treatments have been approved by the Institutional Animal Care and Use Committee at the First Affiliated Hospital of Nanchang University (Animal Welfare Assurance).

### Cell culture and transduction of human MSCs

Culture of human endothelial cells (HUVEC) and human bone-marrow derived MSCs were described previously [11]. For generation of adeno-associated vectors, null or antisense for miR-205-5p (as-miR-205-5p) or complete coding sequence for MALAT1 was cloned into a pCMV-DsRed-Express Vector (Catalog number: 632416; Clontech, Mountain View, CA, USA) backbone at the site between CMVp and red fluorescent protein (RFP), as described [11]. The adeno-associated vector production has been described before [24]. AAV serotype 2 was used. The transfection was done using a ratio of 50 for viruses versus cells. The successfully transfected cells expressed RFP, which was used for flow cytometry-based cell purification 48 hours after transfection, as described before [25–27].

### Transwell co-culture and HUVEC tube formation assay

Transwell assay was done as previously described [27]. Briefly, same number of HUVECs (4,800 cells/cm^2^) were seeded in the collagen gel to grow in the lower chamber, while same number of different MSCs (null-MSCs, as-miR-205-5p-MSCs, MALAT1-MSCs) were seeded in the upper chamber. After 3 days’ culture, HUVEC tube formation assay was done, as previously described [4].

### Diabetic mice, DF and MSC transplantation

Diabetes was induced in 12-week-old male immune-deficient NOD/SCID mice (SLAC Laboratory Animal, Shanghai, China) by single intraperitoneal injection of 130 mg/kg streptozotocin (STZ), as described before [26]. One week later, the mice that had developed diabetes received induction of foot ulcer (and) transplantation of MSCs, as described [24]. Fasting blood glucose has been described before [28]. Beta cell mass measurement has been described before [29]. For vessel density measurement, 50 μl FITC-lectin (Vectorlabs, Burlingame, CA USA) was injected via tail vein to the mice shortly before sacrifice. The vessel density was determined as the ratio of lection+ area versus total measured area, as described before [11].

### Quantitative real-time PCR (RT-qPCR)

Total RNA was extracted using the miRNeasy mini kit (Qiagen, Hilden, Germany). Complementary DNA preparation and quantitative real-time PCR (RT-qPCR) were routinely done, using primers (miR-205-5p, MALAT1, VEGF) purchased from Qiagen. Data were collected and analyzed using 2-△△Ct method. Values of genes were first normalized against GAPDH, and then compared to experimental controls.

### Histology and immunostaining

After FITC-lectin perfusion, mouse pancreas or limb skin tissue was dissected out and fixed in 4% paraformaldehyde (PFA, Sigma-Aldrich, St. Louis, MO, USA) for 6 hours. After overnight incubation in 30% sucrose, samples were frozen in liquid nitrogen and embedded in tissue freezing medium. Fluorescent immunostaining was done as described before [11]. FITC-lectin was detected by direct green fluorescence. Insulin and glucagon staining used guinea pig polyclonal antibody against insulin (1:500, Ab7842, Abcam, Cambridge, MA, USA) and monoclonal antibody against glucagon (1:400, Ab10988, Abcam), respectively. Hoechst 33342 (HO, Sigma-Aldrich) staining was done for visualizing nucleus.

### ELISA

The cellular protein was extracted by RIPA buffer, after which cellular VEGF was determined using a human VEGF enzyme-linked immunosorbent assay (ELISA; DVE00; R&D System, Los Angeles, CA, USA), as described before [11].

### Statistical analysis

All values represent the mean ± standard deviation (SD). Statistical analysis of group differences was carried out using a one-way analysis of variance (ANOVA) test followed by the Fisher’s Exact Test to compare two groups (GraphPad Software, Inc. La Jolla, CA, USA). A value of p<0.05 was considered statistically significant after Bonferroni correction.
